# Effect of non-random mating on genomic and BLUP selection schemes

**DOI:** 10.1186/1297-9686-44-11

**Published:** 2012-04-11

**Authors:** Kahsay G Nirea, Anna K Sonesson, John A Woolliams, Theo HE Meuwissen

**Affiliations:** 1 Department of Animal and Aquacultural Sciences, Norwegian University of Life Science, Norwegian, P.O. Box 5003 N-1432 Ås, Norway; 2 Nofima AS, Norwegian, P.O. Box 5010 1432 Ås, Norway; 3 The Roslin Institute and Royal (Dick) School of Veterinary Studies, University of Edinburgh, Easter Bush Campus, Midlothian, EH25 9RG, Scotland, UK

## Abstract

**Background:**

The risk of long-term unequal contribution of mating pairs to the gene pool is that deleterious recessive genes can be expressed. Such consequences could be alleviated by appropriately designing and optimizing breeding schemes i.e. by improving selection and mating procedures.

**Methods:**

We studied the effect of mating designs, random, minimum coancestry and minimum covariance of ancestral contributions on rate of inbreeding and genetic gain for schemes with different information sources, i.e. sib test or own performance records, different genetic evaluation methods, i.e. BLUP or genomic selection, and different family structures, i.e. factorial or pair-wise.

**Results:**

Results showed that substantial differences in rates of inbreeding due to mating design were present under schemes with a pair-wise family structure, for which minimum coancestry turned out to be more effective to generate lower rates of inbreeding. Specifically, substantial reductions in rates of inbreeding were observed in schemes using sib test records and BLUP evaluation. However, with a factorial family structure, differences in rates of inbreeding due mating designs were minor. Moreover, non-random mating had only a small effect in breeding schemes that used genomic evaluation, regardless of the information source.

**Conclusions:**

It was concluded that minimum coancestry remains an efficient mating design when BLUP is used for genetic evaluation or when the size of the population is small, whereas the effect of non-random mating is smaller in schemes using genomic evaluation.

## Background

Selection and parent mating patterns are the two major components of a breeding program and must be optimised with respect to genetic gain (ΔG) and rate of inbreeding (ΔF). In the literature, several selection and mating designs have been reported that aim at reaching a high ΔG and/or low ΔF [1,2], since a high ΔF represents a risk for the long-term success of breeding programs. While there is a consensus on selection procedures [3,4], many mating designs have been developed, mostly aimed at avoiding the mating of parents that are more related than average [1,5-7].

Factorial mating consists in producing half sibs from male and female parents at the expense of full sibs, while preserving offspring numbers per parent [8]; with minimum coancestry mating (MC), relationships between mating pairs are minimized to minimize progeny inbreeding [9]; with compensatory mating, parents with a small number of selected sibs are preferably mated to those with a large number of selected sibs [10]. More recently, Henryon *et al. *[2] considered mating animals by minimizing the covariance between ancestral contributions (MCAC), which allows the impact of future changes in ancestral contributions to be less mutually dependent and favours the selection of animals with optimum contributions from their ancestors. All these mating schemes have shown some benefits in increased genetic gain and/or reduced inbreeding but comparisons reported in the literature have not concluded on any clearly superior mating design, partly because only a limited number of mating designs were considered in each of the comparisons.

All studies mentioned above have used evaluation methodologies that rely on phenotype alone or combined with pedigree to provide best linear unbiased prediction (BLUP) estimated breeding values (EBV). Recently, the concept of genomic evaluation has been introduced in animal breeding [11], in which the prediction of the total genetic value of selection candidates is based on dense genotyping data and estimates of SNP effects, which are calculated from a genotyped and phenotyped training set of individuals from the population under selection. One of the reported benefits of this approach is that the accuracy of EBV increases thanks to an improved prediction of the Mendelian sampling term. The consequence is a potential reduction of ΔF in selection schemes [12] because it reduces the emphasis put on family information and increases that on the individual's own merit. Hence, it is time to re-examine the benefits of non-random mating given the methodological developments in genomic selection (GS).

Therefore, this study aims to assess the interaction between genetic evaluation methodology and non-random mating designs and their impact on ΔF and ΔG. It compares the impacts of pair-wise versus factorial family structures and performance-testing versus sib-testing, which differ in their information source, the candidate and sibs of the candidate, respectively. Such structure and testing designs are common variants in aquaculture breeding schemes.

## Methods

### Populations

We simulated a Fisher-Wright population with an effective population size of 1000 and 4000 generations to construct a base generation G0 for use in subsequent selection. The population had a diploid genome with a total length of 10 Morgan with 10 chromosomes, each 1 Morgan long. SNP mutations were introduced in all generations used to generate G0 at a rate of 10^-8 ^per base pair per meiosis, assuming

1 000 000 base pairs per cM. Parent to offspring transmission of SNP followed Mendelian inheritance. A period of 4 000 generations has been shown to be sufficient to achieve mutation-drift equilibrium [13]. In each of the 4 000 generations, n_m _= 500 males and n_f _= 500 females were produced by random selection (with replacement) of a male and a female parent. The same procedure was repeated 100 times to create 100 replicates.

In G0, 200 male and 200 female candidates were randomly sampled, i.e. n_m _= n_f _= 200, and, if appropriate, another sample of identical size was randomly chosen as test sibs. From G0 until G12, n_s _sires and n_d _dams were selected and mated according to the procedures described below. Each mating produced 2n_o _offspring per mating pair, equally divided between males and females, where n_s _= n_d _= 25 (or 50), with 2n_o _= 2, 8 or 16 depending on the family structure (see below). The total number of candidates in each generation G1 to G12 was 400, equally divided between males and females, with an additional 400 if required for sib testing. In aquaculture breeding programs, the fixed cost of conducting a test is an important resource constraint, rather than the variable cost of producing additional sibs for testing, if testing is necessary.

### Genome, markers and true breeding *v*alues

In G0, a random sample of 1 000 SNP with minor allele frequency (MAF) > 0.05 were considered as quantitative trait loci (QTL). Allelic values were assigned to the QTL by independent sampling of effects from the Laplace distribution. The QTL effects were assumed to be additive and effects were standardized such that the total genetic variance (variance of breeding values) was 10. Phenotypes were also created assuming a heritability of 0.4 or 1. If the heritability was 1, the phenotype was equal to the true breeding value but if it was 0.4, an environmental deviation was drawn independently from N(0, 15) and added to the true breeding value for G0 to G12. Our objective was to track *m *= 5 000 markers from G0 onwards and since more SNP were generated than needed SNP with the highest MAF were selected from those not selected as QTL.

### Breeding schemes: Recording, estimation of breeding values, selection and mating

#### Information source

We studied the effect of selection and mating on ΔF and ΔG with either a performance test of the candidates (CAND) or a sib test (SIB). In the CAND schemes, the candidates were directly recorded for the trait under selection. In the SIB schemes, the full sib families were divided into two sets, i.e. one set was recorded for the trait and one set provided the candidates for selection. This is the case if the trait is for instance a disease challenge or a meat quality assessment resulting in the death of the tested individuals.

#### Evaluation and selection

Selection among the candidates was performed by truncation selection of n_s _sires and n_d _dams based on the EBV provided by genetic evaluation. Two types of genetic evaluation were considered: (1) BLUP, based on pedigree and phenotypes; and (2) GS using a set of 5 000 SNP, which were assumed to have been genotyped on all individuals of a given generation. BLUP evaluations were done according to the standard methodology of Henderson [14]. Genomic evaluation followed the GS-BLUP model [11] for n phenotypes with *m *SNP loci:

$$y=\sum_{\text{j}=1}^{m}{\text{ x}}_{\text{ij }}{\text{g}}_{\text{j}}+e,$$

where *y *is a vector of phenotypes, *X_ij_* is the standardized number of a reference allele (allele "1") for animal *i *and SNP *j *as assessed by SNP i.e.

$${\text{x}}_{\text{ij}}=\frac{{\text{x}}_{\text{ij}}^{*}-2{\text{p}}_{\text{j}}}{\sqrt{2{\text{p}}_{\text{j}}\left(1-{\text{p}}_{\text{j}}\right)}},$$

where *P_j_* is the frequency of allele "1" at SNP j, x*_ij _is the number of alleles "1" genotyped for animal i at SNP_ij_, *g_j_*is the effect of allele "1" at locus j, and *e *is the vector of random errors assumed to be drawn from N. The elements x_ij _form the incidence matrix **X**. The SNP effects were estimated as

$$\left[X^{\prime }*X+\text{λ}*I\right]\left[\hat{g}\right]=\left[X^{\prime }*y\right].$$

From the above mixed model equation *g *is the vector of estimated effects at the marker loci, and $\text{λ}=m\frac{{\text{σ}}_{\text{e}}^{2}}{{\text{σ}}_{\text{A}}^{2}}$. The variance of each marker effect was assumed to be ${\text{σ}}_{\text{i}}^{2}=\frac{{\text{σ}}_{\text{A}}^{2}}{m}$.

Finally, the genomic EBV (***û***) for selection candidate i was estimated as

$${û}_{i}=\overset{m}{\underset{\text{j}=1}{\sum }}{\text{x}}_{\text{ij}}\;\;{\hat{\text{g}}}_{\text{j}}$$

### Family structure

Two family structures were studied:

(1) The first was a pair-wise family structure (PAIR), where identical numbers of sires and dams, i.e. n_s _= n_d _= 25 (or 50), were selected every generation and each sire was only mated to one dam, to produce 25 (or 50) full-sib families. Each full-sib family had 2n_o _= 16 (or 8) offspring, with an equal sex ratio. Thus, a total of 400 candidates were produced every generation. For the SIB schemes, the 2n_o _offspring were doubled to produce test sibs

(2) The second structure was a factorial family structure (FAC), where identical numbers of sires and dams, i.e. n_s _= n_d _= 25 (or 50), were selected every generation and mated so that each sire was mated with 8 (or 4) dams and each dam was mated with 8 (or 4) sires, producing 200 full-sib families, with 2n_o _= 2, with 25 (or 50) paternal half- sib families and 25 (or 50) maternal half-sib families.

### Mating design

Three mating designs were studied:

(1) Random mating (RAND), in which selected sires and dams were paired by random sampling without replacement from the candidates

(2) Minimum coancestry mating (MC), where the sets of male and female mating pairs were chosen in order to minimize the average coancestry between the mates, as calculated from the pedigree. Randomly-chosen parents were swapped until a decrease in coancestry was observed, at which point the swapped parents were retained, as in [5]. This was repeated until no more improvement was achieved.

(3) Minimized covariance of ancestral contributions mating (MCAC), in which contributions of each ancestor to each candidate were calculated and the covariance between the contributions for the proposed set of male and female mating pairs was minimised [2]. Starting from the random mating design, parents were randomly chosen and swapped until the MCAC mating criterion improved and then the swapped parents were retained. The process was stopped when no further improvement of MCAC was achieved.

### Simulated schemes

The three mating designs (RAND, MC and MCAC) were tested for all combinations of evaluations (BLUP or GS), family structures (PAIR or FAC), information sources (CAND or SIB), and h^2 ^(0.4 or 1). This amounts to a total of 48 comparisons. An additional comparison was made considering an increased number of parents (n_s _and n_d_).

### Statistics for assessment

For each scheme, ΔG and ΔF were calculated. ΔF was computed as the negative of the slope of the regression of ln(1-F_t_) on t for F_6_-F_12 _where F*_t_*is the level of inbreeding at generation t, ΔG was computed as the difference in genetic level between G12 and G6 divided by 6. The use of generations 6 to 12 made it possible to reach a near-Bulmer equilibrium and to stabilize the degree of assortative mating. Each simulation was replicated 100 times and the replicates were averaged. Standard errors of the mean were calculated from the variance between replicates.

## Results

**Figure 1:**
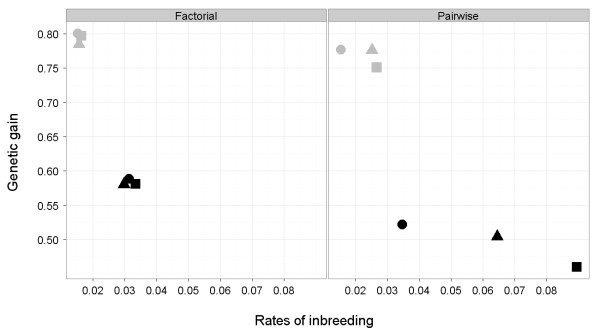
**Interactions between genetic gain and rates of inbreeding in different scenarios**. RAND or random mating (□), MC or minimum coancestry (○) and MCAC or minimizing covariance of ancestral contributions mating (△) mating designs with black color for the BLUP/SIB scheme: where n_s _= 25 and n_d _= 25 parents were selected on BLUP EBV using sib information; with grey color for the GS/SIB schemes: where n_s _= 25 and n_d _= 25 parents were selected on GW EBV using sib information; the family structure of the breeding schemes was either factorial (FAC) or pair wise (PAIR), h^2 ^= 0.4.

Tables 1, 2 and 3 present rates of inbreeding (ΔF) and genetic gain (ΔG) generated under different evaluation methods (GS or BLUP), information sources (CAND or SIB), family structures (FAC or PAIR), and mating designs (RAND, MC and MCAC) for discrete generations with an initial heritability of 0.4 or 1. Figure 1 shows a summary of the results.

**Table 1:** Effect of mating when family structure, information source and genetic evaluation vary and h^2 ^= 0.4

Scheme	Mating	Pair-wise family structure		Factorial family structure	
		
		ΔF	ΔG	ΔF	ΔG
BLUP/CAND	RAND	4.09	0.684	2.02	0.731

	MC	1.98	0.723	2.02	0.766

	MCAC	3.41	0.741	1.84	0.741

BLUP/SIB	RAND	8.95	0.460	3.34	0.581

	MC	3.47	0.522	3.13	0.589

	MCAC	6.45	0.504	2.98	0.580

GS/CAND	RAND	2.45	0.780	1.58	0.829

	MC	1.52	0.814	1.55	0.813

	MCAC	2.39	0.813	1.49	0.841

GS/SIB	RAND	2.67	0.751	1.64	0.797

	MC	1.54	0.777	1.53	0.801

	MCAC	2.52	0.776	1.57	0.785

Rates of inbreeding in percentage (ΔF) and genetic gain in genetic standard deviation units (ΔG) generated per generation when n_s _= 25, n_d _= 25 and h^2 ^= 0.4 for schemes where parent selection is on BLUP EBV using candidate information (BLUP/CAND) or using sib information (BLUP/SIB); for schemes where parent selection is on GS EBV using candidate information (GS/CAND) or sib information (GS/SIB); RAND: random; MC: minimum coancestry; MCAC: minimizing covariance of ancestral contributions mating; standard errors of means of 100 replicates of genetic gain were less than 0.38% and of rates of inbreeding were less than 0.11%

**Table 2:** Effect of mating when family structure and genetic evaluation vary and h^2 ^= 1

Scheme	Mating	Pair-wise family structure		Factorial family structure	
		
		ΔF	ΔG	ΔF	ΔG
BLUP/SIB	RAND	1.95	0.885	1.37	0.939

	MC	1.32	0.985	1.45	0.985

	MCAC	2.07	0.945	1.28	0.909

GS/SIB	RAND	1.90	0.894	1.36	0.928

	MC	1.33	0.928	1.33	0.950

	MCAC	2.04	0.916	1.29	0.951

Rates of inbreeding in percentage (ΔF) and genetic gain in genetic standard deviation units (ΔG) generated per generation when n_s _= 25, n_d _= 25 and h^2 ^= 1 for schemes where parent selection is on BLUP EBV using sib information (BLUP/SIB); for schemes where parent selection is on GS EBV using sib information (GS/SIB); RAND: random; MC: minimum coancestry; MCAC: minimizing covariance of ancestral contributions mating; standard errors of means of 100 replicates of genetic gain were less than 0.21% and of rates of inbreeding were less than 0.38%

**Table 3:** Effect of mating when selection intensity changes with a pair-wise family structure

Scheme	Mating	ΔF	ΔG
BLUP/SIB	RAND	0.0123	0.494

	MC	0.0114	0.496

	MCAC	0.0106	0.488

GS/SIB	RAND	0.0068	0.648

	MC	0.0060	0.659

	MCAC	0.0038	0.646

Rates of inbreeding in percentage (ΔF) and genetic gain in standard deviation units (ΔG) generated per generation when n_s _= 50, n_d _= 50 and h^2 ^= 0.4; standard errors of means of 100 replicates of genetic gain were less than 0.27% and of rates of inbreeding were less than 0.1%

### Effect of genetic evaluation methods

The effect of the genetic evaluation method was obtained by comparing the ΔF and ΔG generated under GS/CAND and BLUP/CAND, GS/SIB and GS/CAND schemes (Table 1) using the RAND mating design. For PAIR family structures, a 0.016 (40%) reduction in ΔF and a 0.096 (14%) increase in ΔG were observed when candidate records were used for GS evaluation compared to BLUP evaluation. There was a 0.063 (70%) reduction in ΔF and a 0.291 (60%) increase in ΔG when sibs of the candidates were recorded for genetic evaluation (Table 1). For FAC family structures, a 0.004 (9%) reduction in ΔF and a 0.098 (13%) increase in ΔG were observed when the candidates were recorded and a 0.017 (51%) reduction in ΔF and a 0.216 (37%) increase in ΔG when sibs of the candidates were recorded (Table 1). The pattern was similar when the intensity of selection and heritability were increased (Results not shown). Similar results were reported in [13,15], in which sib records were used for BLUP and GS evaluation.

### Effect of information sources

The effect of information sources for schemes with BLUP and GS evaluation is presented in Table 1. The results show that non-random mating designs reduced ΔF by a greater factor for schemes with SIB information than for those with CAND information. For example, MC had a lower ΔF than RAND under the BLUP/CAND (0.021 i.e. 52% higher) and the BLUP/SIB (0.055 i.e. 61%) schemes. Similarly, MCAC had a lower ΔF than RAND under the BLUP/CAND (0.007 i.e. 17%) and the BLUP/SIB (0.025 i.e. 28%) schemes. Hence, these results show that a larger reduction in ΔF was generated by MC mating compared to MCAC mating.

ΔG was always higher in schemes with CAND information compared to schemes with SIB information and MC and MCAC had a higher ΔG than RAND. For example, MC had a higher ΔG than RAND for the BLUP/CAND (0.057 i.e. 6%) and the BLUP/SIB (0.062 i.e. 13%) schemes; MCAC also had a higher ΔG than RAND for the BLUP/CAND (0.057 i.e. 8%) and the BLUP/SIB (0.044 i.e. 10%) schemes.

The differences in ΔF between schemes with GS evaluation were smaller than those with BLUP evaluation, irrespective of the information source. As shown in Table 1, MC and MCAC had a lower ΔF than RAND for the GS/CAND (respectively, 0.009 i.e. 38% and 0.001 i.e. 2%) and the GS/SIB schemes (respectively, 0.011 i.e. 42% and 0.002 i.e. 6%).

MC and MCAC generated somewhat higher ΔG than RAND: 0.033 i.e. 4% for the GS/CAND scheme and 0.025 i.e. 3% for the GS/SIB scheme. ΔG was always higher in the schemes with CAND information than in schemes with SIB information. However, compared to the ΔG generated by RAND, higher rates of ΔG were observed by MC and MCAC, especially in the schemes with SIB information. These results show that mating designs such as MC and MCAC can generate higher ΔG when BLUP evaluation uses sib information compared to candidate information.

In general, compared to schemes with BLUP evaluation the effect of information source (CAND or SIB) was smaller in schemes with GS evaluation (Table 1).

### Effect of family structures

The impact of family structure (PAIR or FAC) on ΔF and ΔG was assessed by comparing schemes with BLUP evaluation in Table 1. The effect of MC and MCAC on ΔF was larger in the schemes with a PAIR family structure than in those with a FAC family structure. For instance, under the BLUP/SIB scheme, MC and MCAC had a lower ΔF (respectively, 0.055 i.e. 61% 0.025 i.e. 28%) than RAND in schemes with a PAIR structure; whereas, in schemes with a FAC structure, MC and RAND had a similar ΔF and MCAC had a lower ΔF (0.002 i.e. 6%). Likewise, MC and MCAC had a higher ΔG than RAND in schemes with a PAIR structure (respectively, 0.062 i.e. 13% and 0.44 i.e. 10%) but in schemes with a FAC structure, MC had a higher ΔG than RAND (0.008 i.e. 1%) and MCAC had a similar ΔG.

The impact of family structure on ΔF for schemes with GS evaluation was evaluated by comparing schemes with GS evaluation in Table 1. In non-random mating designs, ΔF was lower for schemes with a FAC structure than for schemes with a PAIR structure. For instance, comparisons for the GS/SIB schemes shows that MC and MCAC led to a lower ΔF than RAND in the schemes with a PAIR structure (respectively, 0.011 i.e. 42% and 0.002 i.e. 6%) and with a FAC structure (respectively, 0.001 i.e. 7% and 0.001 i.e. 4%). Similarly, comparisons for the GS/SIB schemes show that MC and MCAC had a higher ΔG than RAND in the scheme with a PAIR structure (respectively, 0.026 i.e. 3% and 0.025 i.e. 3%) but in the scheme with a FAC structure, MC had a higher ΔG (0.004 i.e. 1%), while MCAC had a lower ΔG (0.012 i.e. 2%). Similar contrasts were observed for the BLUP/CAND and the GS/CAND schemes. Thus, these results show that the mating designs consistently reduced ΔF and increased ΔG more for schemes with a PAIR structure than for schemes with a FAC structure. In addition, for schemes with a PAIR structure, the effect of mating design was higher with BLUP evaluation than with GS evaluation. Also, the effect of mating designs decreased when the selection intensity decreased (see Tables 1, 2 and 3).

### Effect of heritability

The results indicate that increasing heritability generally slightly decreased ΔF but substantially increased ΔG for both family structures. For example, when heritability was increased from 0.4 to 1 for the BLUP/SIB schemes with a PAIR family structure, RAND, MC and MCAC had a lower ΔF (respectively, 0.070 i.e. 78%, 0.022 i.e. 62% and 0.044 i.e. 68%) and a higher ΔG (respectively, 0.425 i.e. 92%, 0.463 i.e. 89% and 0.441 i.e. 88%) (Tables 1 and 2). The same comparisons with a FAC family structure are shown in Tables 1 and 2. Similarly, for the GS/SIB schemes, when heritability was increased from 0.4 to 1, RAND, MC and MCAC had a lower ΔF (respectively, 0.008 i.e. 29%, 0.002 i.e. 14% and 0.005 i.e. 19%) and a higher ΔG (respectively, 0.143 i.e. 19%, 0.151 i.e. 19% and 0.14 i.e. 18%) (Tables 1 and 2). Similar results were obtained for the BLUP/CAND and the GS/CAND schemes (Results not shown). Heritability had a similar effect on ΔF and ΔG when the intensity of selection was increased (Results not shown). Overall, increases in ΔG due to increasing heritability were larger for schemes with a PAIR structure than for schemes with a FAC structure.

## Discussion

This study was designed mainly for aquaculture breeding schemes. However, the conclusions can be extrapolated to any domesticated species. We examined the effect of non-random mating when genetic evaluation, information source, family structure and heritability varied. The approach maintained a constant intensity among candidates with truncation selection and examined differences in ΔF and ΔG. The primary findings were that non-random mating designs had less impact on schemes with GS evaluation than on schemes with BLUP evaluation, and that this impact was greater for schemes with a PAIR structure than for schemes with a FAC structure and also greater for schemes with SIB information than for schemes with CAND information (Figure 1). We also compared MC with MCAC, a novel method proposed by Henryon *et al. *[2], but found that differences between these mating designs were small, with the balance of evidence pointing to MC as being more effective.

Among the different scenarios, the greatest effects of non-random mating, both for MC and MCAC, were a reduction of ΔF rather than an increase of ΔG. Selection intensity (*i*) was held constant, with truncation selection, and in such schemes the primary selective advantage is the individual's breeding value or its components (the EBV and prediction error) [16]. In such a system, ΔF increases with *i^2 ^*[17], thus one can expect that if ΔF instead of *i *had been held constant across schemes, then substantial benefits in ΔG would have emerged for the schemes with a low ΔF. This is because *i *would need to be reduced in schemes with a greater ΔF, and thus ΔG would decrease as it is directly proportional to *i*. This dependence of ΔF on *i *explains why more than two-fold increases in ΔF were observed when the number of parents per sex was halved (cf. Tables 1, 3). If the comparisons were performed with optimum contribution selection [3], then a broadly similar outcome may be anticipated, although some differences may occur as the selective advantage with optimum contributions is the estimated Mendelian sampling term [18] distinct from truncation selection where the selective advantage is the individual's breeding value or its components [16].

The outcomes of two of the dimensions of this study, GS versus BLUP and CAND versus SIB are driven by the increased accuracy of estimation of Mendelian sampling terms. Increased accuracy of Mendelian sampling terms in truncation selection reduces ΔF primarily by reducing the importance of inherited selective advantages that are conferred by parents to offspring, as parental EBV have less weight in the selection decisions made on candidates. In the context of genomic evaluation and BLUP, the additional accuracy and impact on ΔF have been highlighted by Daetwyler *et al. *[12]. The use of sib-testing is an extreme example, whereby all information for selection among the candidates is derived from their sibs; this sib information helps to increase the accuracy of the EBV of the sire and the dam, and through them the candidate, but provides no direct information on the candidate's Mendelian sampling term. Hence the benefit in ΔF from using Cand versus SIB is smaller since the candidate's own performance does allow an estimate of the Mendelian term.

A heritability equal to 1 was included in this study to explore the impact of increased evaluation accuracy in schemes with SIB information. In schemes with CAND information, h^2 ^= 1 is the same as mass selection for both BLUP and GS and the impact of non-random mating on mass selection has been explored by Caballero *et al. *[6]. For schemes with SIB information, the contrast between BLUP and GS is strongest when h^2 ^= 1, since with BLUP the lack of information on Mendelian sampling terms of candidates remains, but with GS the accuracy of Mendelian sampling term estimates is expected to be high. This impact was observed, with substantial increases in ΔG, and with the greatest reductions in ΔF occurring in schemes using genomic evaluations rather than BLUP, for which the reductions in ΔF were relatively small. In the schemes using genomic selection, the number of animals tested in genomic evaluation was not large enough to achieve an accuracy of 1. However, it is plausible that such accuracy could be achieved in the future. In this case, the outcome would be different from those simulated in this study as there would be no need for the SIB testing, and then both CAND and SIB testing would proceed as if it was mass selection by truncation on a recorded phenotype with h^2 ^= 1. In this case, there would still be potential to reduce ΔF for the same ΔG by using a FAC family structure, by using MC and other non-random mating schemes, and by using optimal contribution selection [19].

Schemes with a FAC structure are mating designs that do not depend on non-randomness, neither in relation to pedigree nor EBV. Such schemes consistently generated lower ΔF compared to schemes with a PAIR structure (Table 1). These results are in agreement with almost all previous studies [2,19] where lower ΔF were generated in schemes with a FAC structure compared to schemes with a hierarchal family structure, and extend this result to schemes using genomic evaluation. One published exception to this finding of a benefit from a FAC family structure was in aquaculture [20], when restrictions were placed on family tanks (i.e. full-sib families) but the results of that study are ambiguous and need further clarification. The benefit of FAC family structures has been shown to result from the reduced variance in selective advantages by reducing the component of variance due to mates [21]. This is achieved by 'averaging' mate effects over a greater number of individuals. This is in contrast to PAIR family structures, where the selective advantages of the mates are completely confounded. Future selection decisions should reduce the deviation of long-term contributions of the ancestors from their optimal contributions. Relative to RAND mating, non-random mating designs substantially reduced ΔF in schemes with a PAIR structure, but the differences were minor in the schemes with a FAC structure, most likely due to the improved structure already conferred by FAC.

MC and MCAC both aim at improving family structure for selection but use different approaches: MCAC chooses mates to minimize the covariance of ancestral contributions, whereas MC minimizes the expected variance of contributions of the ancestors for a randomly chosen offspring. The latter is because the sum of squared contributions of the ancestors weighted by their within-family variance equals the diagonal of the relationship matrix, i.e. 1 + F of the offspring. Thus minimizing F by MC mating is approximately equivalent to minimising the sum of squares of contributions, and thus the variance of the contributions of an individual, since the mean contribution is not affected by the mating scheme. The justification for MCAC is that lower covariance among ancestral contributions allows future selection to shift individual contributions towards optimum values [18,21], with less cost in inbreeding per unit change. In contrast, the smaller variance among ancestral contributions that is targeted by MC helps to minimize their changes when some offspring are selected. With the simulated schemes, the results suggest that minimizing the variance of contributions per offspring is more effective than minimizing the sum of covariances of ancestral contributions over all of the offspring. This could justify the use of MC instead of MCAC.

## Conclusions

This study examined the benefits of non-random mating in simulated schemes with different scenarios. The conclusion is that non-random mating is more beneficial when the evaluation is based upon sib test records than candidate records. Changing the family structure from pair-wise to factorial was always beneficial in reducing rates of inbreeding but reduced the benefits from non-random mating per se. In this study, non-random mating was shown to be more beneficial with BLUP evaluation than with genomic selection.

## Competing interests

The authors declare that they have no competing interests.

## Authors' contributions

Kahsay G Nirea wrote the draft manuscript and ran the computer programs. Theo HE Meuwissen and Anna K Sonesson wrote simulation computer programs. Anna K Sonesson, John A Woolliams and Theo HE Meuwissen edited the drafted manuscript. Finally, all authors have approved the final manuscript for publication.
